# P-723. High Prevalence of Chemsex in PrEP Users in Buenos Aires, Argentina

**DOI:** 10.1093/ofid/ofaf695.934

**Published:** 2026-01-11

**Authors:** Isabel Pastor, Marina Alonso Serena, Mariana Angelica Kundro, Guillermo Alberto Viloria, Maria Belen Zorz, Federico Cardozo, Diego Cecchini, Jose Barletta, Iael Altclas, Marcelo H Losso

**Affiliations:** Hospital JM Ramos Mejía, Buenos Aires, Ciudad Autonoma de Buenos Aires, Argentina; Hospital JM Ramos Mejía, Buenos Aires, Ciudad Autonoma de Buenos Aires, Argentina; Hospital General de Agudos José María Ramos Mejía, Ciudad Autonoma de Buenos Aires, Ciudad Autonoma de Buenos Aires, Argentina; Hospital General de Agudos José María Ramos Mejía, Ciudad Autonoma de Buenos Aires, Ciudad Autonoma de Buenos Aires, Argentina; Hospital General de Agudos José María Ramos Mejía, Ciudad Autonoma de Buenos Aires, Ciudad Autonoma de Buenos Aires, Argentina; Hospital General de Agudos José María Ramos Mejía, Ciudad Autonoma de Buenos Aires, Ciudad Autonoma de Buenos Aires, Argentina; Hospital General de Agudos Dr. Cosme Argerich, Ciudad Autonoma de Buenos Aires, Ciudad Autonoma de Buenos Aires, Argentina; Hospital General de Agudos Dr. Juan A. Fernández, Ciudad Autonoma de Buenos Aires, Ciudad Autonoma de Buenos Aires, Argentina; Hospital Donación Francisco Santojanni, Ciudad Autonoma de Buenos Aires, Ciudad Autonoma de Buenos Aires, Argentina; Hospital JM Ramos Mejía, Buenos Aires, Ciudad Autonoma de Buenos Aires, Argentina

## Abstract

**Background:**

The practice of chemsex refers to the intentional use of psychoactive substances to enhance sexual activity or pleasure. Pre-exposure Prophylaxis (PrEP) for HIV consists in the use of antiretroviral drugs to reduce the risk of infection. A history of having practised chemsex has been associated with a higher frequency of sexually transmitted infections (STIs) diagnosis among PrEP users, probably due to the likelihood of engaging in high-risk behaviors. In Argentina, there is limited information regarding this issue. This research aims to characterize the practice of chemsex and STIs prevalence in PrEP users in four hospitals in Buenos Aires, Argentina.

**Methods:**

This is a descriptive, cross-sectional study. The medical records and a self-administered ad-hoc questionnaire were used to obtain the information.

**Results:**

From February to October 2024, 165 PrEP users were included in the study. The prevalence of chemsex, during the last year, was 32%. The drugs used, in order of frequency, were: MDMA (ecstasy), 21 %; cocaine, 14 %; gamma-hydroxybutyrate (GHB), 12 %; ketamine, 10 %; methamphetamine, 6 %; mephedrone, 2 %; and gamma-butyrolactone (GBL), 1 %. Sixty percent reported the use of 2 or more drugs simultaneously. The sexualized use of marijuana, poppers, sildenafil/tadalafil and/or alcohol was also investigated, with a prevalence of 73%, during the same period. We found an association between the practice of chemsex and a higher number of sexual partners (p 0.0008), group sex (p 0.002) and diagnosis of at least one STI during the previous year (p 0.004), primarily due to urethritis and/or proctitis (p 0.007). No HIV infections were reported.

**Conclusion:**

We found a high prevalence of chemsex among PrEP users in Buenos Aires, Argentina. This practice was associated with higher prevalence of STIs and high-risk behaviours that may be associated with increased transmission. These findings should inform PrEP and sexual health policymaking.

**Disclosures:**

All Authors: No reported disclosures

